# Why Do We Not Assess Sympathetic Nervous System Activity in Heart Failure Management: Might GRK2 Serve as a New Biomarker?

**DOI:** 10.3390/cells10020457

**Published:** 2021-02-21

**Authors:** Leonardo Bencivenga, Maria Emiliana Palaia, Immacolata Sepe, Giuseppina Gambino, Klara Komici, Alessandro Cannavo, Grazia Daniela Femminella, Giuseppe Rengo

**Affiliations:** 1 Department of Translational Medical Sciences, University of Naples “Federico II”, 80131 Napoli, Italy; leonardo.bencivenga@unina.it (L.B.); mariaemiliana.palaia@unina.it (M.E.P.); immacolata.sepe@unina.it (I.S.); giuseppina.gambino@unina.it (G.G.); alessandro.cannavo@unina.it (A.C.); graziadaniela.femminella@unina.it (G.D.F.); 2 Department of Advanced Biomedical Sciences, University of Naples “Federico II”, 80131 Napoli, Italy; 3 Department of Medicine and Health Sciences, University of Molise, 8610 Campobasso, Italy; klara.komici@unimol.it; 4 Istituti Clinici Scientifici Maugeri SPA, Società Benefit, IRCCS, Istituto Scientifico di Telese Terme, 82037 Telese Terme, Italy

**Keywords:** heart failure, cardiac adrenergic nervous system, biomarkers, GRK2, β-adrenergic receptor signaling, Lymphocyte

## Abstract

Heart failure (HF) represents the end-stage condition of several structural and functional cardiovascular diseases, characterized by reduced myocardial pump function and increased pressure load. The dysregulation of neurohormonal systems, especially the hyperactivity of the cardiac adrenergic nervous system (ANS), constitutes a hallmark of HF and exerts a pivotal role in its progression. Indeed, it negatively affects patients’ prognosis, being associated with high morbidity and mortality rates, with a tremendous burden on global healthcare systems. To date, all the techniques proposed to assess the cardiac sympathetic nervous system are burdened by intrinsic limits that hinder their implementation in clinical practice. Several biomarkers related to ANS activity, which may potentially support the clinical management of such a complex syndrome, are slow to be implemented in the routine practice for several limitations due to their assessment and clinical impact. Lymphocyte G-protein-coupled Receptor Kinase 2 (GRK2) levels reflect myocardial β-adrenergic receptor function in HF and have been shown to add independent prognostic information related to ANS overdrive. In the present manuscript, we provide an overview of the techniques currently available to evaluate cardiac ANS in HF and future perspectives in this field of relevant scientific and clinical interest.

## 1. Introduction

Heart failure (HF) represents a major cause of mortality and morbidity worldwide, affecting about 1% to 2% of the global population, with a trend showing a further increase in cases over the next decade [1,2]. The incidence rate is rapidly rising, especially in older patients, so that, according to some authors, HF should be counted among the geriatric syndromes, with a dramatic burden on national health systems [3,4].

HF constitutes a heterogeneous clinical syndrome, the end-stage condition of several structural and functional cardiovascular diseases leading to a myocardial-enhanced pressure load and/or reduced ventricular output. The current European Society of Cardiology (ESC) guidelines establish three forms of HF according to left ventricular ejection fraction (LVEF), echocardiographic features and diagnostic biomarkers: HF with reduced LVEF (HFrEF), mid-range HF (HFmEF), and HF with preserved LVEF (HFpEF) [5]. HFpEF and HFrEF are increasingly considered as two different physiopathological conditions, with wide discrepancies across their epidemiological and etiological profiles that are also reflected in the distinct therapeutic approaches.

Dyspnea, fatigue, and swelling of the sloping areas are the typical symptoms and signs of HFrEF, which are caused by peripheral hypoperfusion and fluid retention that occur consequently to heart pump failure. The subsequent activation of the neurohormonal system supports a short-term period of cardiac compensation but progressively turns out to be deleterious, as it involves hyperactivity of the adrenergic nervous system (ANS) that provides increasing levels of circulating catecholamines (CAs), and hyperactivation of the renin–angiotensin–aldosterone system (RAAS). Such a dysregulation of the neurohormonal systems exerts a pivotal role in HF progression and negatively affects patients’ prognosis, determining higher morbidity and mortality rates [6]. Indeed, chronic ANS overdrive and RAAS activation promote myocardial electrical and structural remodeling, cardiac hypertrophy, and fibrosis [7]. Accordingly, β-blockers, angiotensin-converting enzyme inhibitors (ACE-Is)/angiotensin II receptor antagonists (sartans), and mineralocorticoid receptor antagonists (MRAs) have been shown to reduce the mortality in patients with HFrEF across several evidence and represent the first therapeutic line for the management of afflicted patients [5]. Enormous progress has been obtained in the previous decades in the treatment of patients with HFrEF; nevertheless, the prognosis remains poor.

The determination of ANS hyperactivity may bring, in this context, relevant diagnostic and prognostic information, but, to date, all the techniques proposed to assess the cardiac sympathetic nervous system are burdened by intrinsic limits that hinder their implementation in routine clinical practice. The aim of this review is to analyze the current evidence regarding the assessment of ANS hyperactivity in HF patients to point out the opportunity of employing specific molecules involved in beta-receptor signaling as a specific biomarker of adrenergic derangement.

## 2. Sympathetic Nervous System Hyperactivity in HF Pathophysiology

In physiological conditions, the sympathetic nervous system supports cardiac activity through the modulation of dromotropy, cronotropy, inotropy, and lusitropy. Moreover, the balance between the sympathetic and parasympathetic nervous systems regulates the peripheral resistance and cardiac output [8].

**Figure 1:**
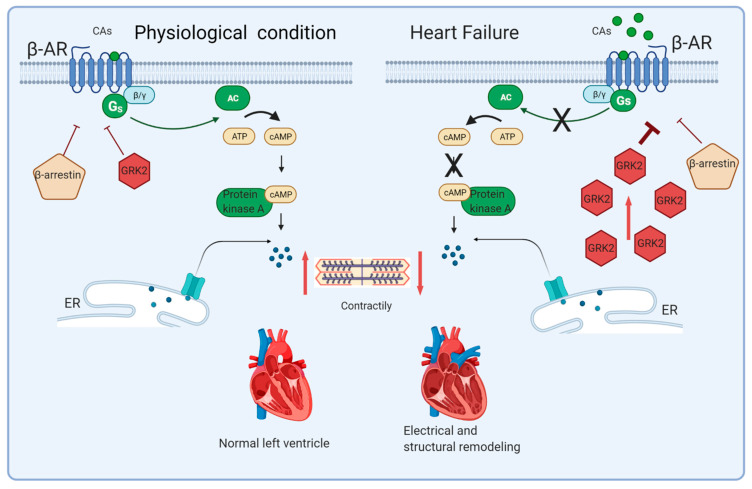
Graphical representation of β-AR signaling in the physiological condition (left) and heart failure (right). In the physiological condition (left panel), β-AR stimulation by CA results in the dissociation of the stimulatory G-protein α-subunit (Gαs) from Gβγ. Gαs stimulates adenylate cyclase (AC) to produce cAMP, which leads to increased contractility by activating the protein kinase A (PKA). The G protein-coupled receptor kinase (GRK2) translocates to the membrane and phosphorylate agonist-bound β-AR, leading to the decoupling of the G protein. Finally, β-arrestins bind the complex, triggering receptor internalization and downregulation. In heart failure (right panel), the increase in CA levels determines the β-AR hyperactivation and consequent GRK2 upregulation, which, in the long term, results in β-AR dysfunction. (AC, adenylate cyclase, ATP, adenosine triphosphate, CAs, catecholamines, β-Ars, β-adrenergic receptors, cAMP, cyclic adenosine monophosphate, ER, endoplasmic reticulum, GRK2, G protein-coupled receptor kinase 2, and Gs, stimulatory G protein). Created with BioRender.com.

The CA-mediated stimulation of β-adrenergic receptors (β-ARs) of the superfamily of G-protein-coupled receptors (GPCRs), initially determines the exchange of guanosine diphosphate (GDP) for guanosine triphosphate (GTP), allowing dissociation of the G-protein and G-alpha subunit to activate adenylate cyclase (AC), which converts adenosine triphosphate (ATP) into cyclic adenosine monophosphate (cAMP). This latter metabolite allows the protein kinase A (PKA) to release free intracellular calcium through both L-type calcium channels of the plasma membrane and ryanodine receptors of the sarcoplasmic reticulum. Furthermore, PKA facilitates sarcoplasmic reticulum calcium reuptake and cardiomyocyte repolarization through stimulation of the sodium pump. Besides producing cardiac muscle contraction by the phosphorylation of many effector substrates, PKA importantly contributes to the uncoupling and desensitization of β-ARs [9] (Figure 1).

Modulation of the adrenergic pathway is crucial for the proper functioning of the cardiovascular system, as β-AR persistent activation causes detrimental dysfunction in the signaling pathway and an inverse effect of depressed cardiac function [10]. Indeed, enhanced stimulation of the ANS constitutes a hallmark of HF, characterized by the augmented release of norepinephrine (NE) and epinephrine (EPI) from both the chromaffin cells of the adrenal gland and heart sympathetic fibers [11].

Three subtypes of β-ARs have been identified in the human heart: β1-AR is the predominant form; its density on the cardiomyocyte membrane is about fourfold more than β2-AR, while the expression of β3-AR is minimal [12]. Interestingly, while the expression of β1-AR is ubiquitous, β2-AR and β3-AR are only present in around 5% of cardiomyocytes; this configuration is reversed in cardiac nonmyocyte cells [13]. Although the stimulation of both β1-AR and β2-AR provides improved cardiac inotropy, lusitropy, and chronotropy, the chronic activity of β2-AR exerts an antiapoptotic effect as opposed to β1-AR, whose persistent stimulation leads to cardiomyocytes apoptosis. Conversely, β3-AR probably counteracts ANS overdrive through the nitric oxide synthase pathway [14].

Activated β-ARs undergo desensitization and downregulation, two key regulatory processes aimed at counteracting damage from excessive CA-mediated stimulation involving GPCR kinase 2 (GRK2) and 5 (GRK5). After being recruited by the Gβγ subunit, these cytosolic serine/threonine enzymes move towards the plasma membrane and phosphorylate C-terminal tail of agonist-binding β-ARs. The phosphorylation of β-AR allows the binding of β-arrestin proteins, which induce the uncoupling of G proteins and prevent further activation of the complex. The phosphorylated and bound β-AR undergoes internalization and, in the case of sustained stimulation, degradation by the lysosome system [15].

As mentioned above, in the failing heart, sympathetic overdrive leads to higher levels of circulating CAs and increased NE spillover from the cardiac sympathetic endings [16]. These phenomena provide chronic hyperstimulation of β-ARs and maladaptive GRK2 upregulation, which, in turn, causes diminished β-AR density on cardiomyocytes through massive desensitization/downregulation processes, with the loss of contractility and inotropic reserve [17]. Accordingly, several models have demonstrated a beneficial effect of GRK2 antagonism in preserving and recovering heart function, and several pieces of literature have focused on GRK2 as an alternative HF therapeutic target [9,18]. GRK2 has also been detected to exert a pivotal role in several signaling pathways, such as interacting in the aldosterone–mineralocorticoid receptor system and β-AR-mediated cardiac insulin resistance [19]. Indeed, it has been involved in several pathophysiological conditions, especially cardiovascular diseases such as hypertension and hypertrophic cardiomyopathy, and metabolic disorders—in particular, metabolic syndrome, type 2 diabetes, and nonalcoholic fatty liver disease [20]. Moreover, the intricate scenario is further complicated by the apparent different mechanisms of action of the β-arrestin isoforms; indeed, the persistent activation of β-arrestin 1 boosts proapoptotic and proinflammatory signaling, thus concurring with HF progression. Indeed, post-myocardial infarction β-arrestin 1 knockout (KO) mice show reduced ischemic area, better cardiac function, and increased survival compared to wild-type mice. Contrarywise, β-arrestin 2 counteracts cell death signaling, thus promoting cell survival, through the regulation of cardiac contractility via sarcoplasmic/endoplasmic reticulum Ca2+-ATPase2a (SERCA2a), modulation of the inflammatory process, and myocyte apoptosis [6].

The hyperactivation of the ANS observed in HF is related to the increased risk of arrhythmias and left ventricular dysfunction, and it represents a pathophysiological prerequisite for therapy with β-blockers [21]. Moreover, higher CAs exposure increases the heart peroxidative metabolism and cardiac oxygen demand, thus contributing to degenerative effects such as necrosis and inflammation, the augmented deposition of collagen, and interstitial fibrosis [8]. Besides, the adrenergic system is also involved in the regulation of several metabolic pathways: ANS overdrive induces alterations in glucose and the lipid metabolism, with the development of insulin resistance and mitochondrial dysfunction [22,23].

## 3. How to Assess Sympathetic Nervous Activity: Methods

Several tools are available to provide an estimation of ANS activity in HF, and these include cardiovascular reflex tests, resting heart rate, heart rate variability (HRV) measures, NE spillover, clinical microneurography, cardiac iodine-123 metaiodobenzylguanidine (123I-MIBG), 11C-hydroxyephedrine (11C-HED) imaging, pupillometry, and blood biomarkers.

The resting heart rate, regulated by the ANS, has been found to exert an independent predictive role on the development of coronary artery disease, as well as on all-cause and cardiovascular mortality. The prognostic value of an increased heart rate in patients with HF has been shown in different clinical trials evaluating heart rate-lowering drugs, such as beta-blockers [24]. More recently, in the BEAUTIFUL trial, a subgroup analysis conducted in patients with resting heart rates higher than 70 bpm showed that heart rate reduction obtained via ivabradine treatment was able to reduce hospitalizations and revascularizations [25].

In physiological conditions, ANS exerts a fine regulation of cardiac and vascular functions through the integration of complex reflex responses, tuned by a balance between the parasympathetic and sympathetic functions. Those reflexes include the arterial baroreceptor reflex, peripheral and central chemoreceptor reflexes, cardiopulmonary mechanosensitive reflex, and pulmonary stretch receptor reflex [26]. In the past decades, Ewing and colleagues developed a battery of five noninvasive bedside tests to assess the ANS function in diabetic patients [27]. The Ewing battery has since been used in different clinical conditions aimed at the evaluation of autonomic function, including in HF [28]. It consists in the assessment of blood pressure and the heart rate response to challenges such as standing, deep breathing, hand grip, and Valsalva. Although easy to perform with minimal equipment, the Ewing battery suffers from low specificity and limited reproducibility.

HRV is the beat-to-beat fluctuation in consecutive heartbeats, and it is regulated by dynamic heart and brain interactions through the ANS branches. The estimation of HRV parameter variations on ECG recordings provides indirect measures of ANS activation in both the time and the frequency domains [29]. In particular, the power of the different frequency bands in the HRV spectra are commonly used as surrogates for parasympathetic and SNS functions. The high-frequency power (HFP) reflects parasympathetic activity, while the low-frequency power (LFP) is modulated by both parasympathetic and sympathetic systems. The LFP/HFP ratio is considered a measure of sympathetic/vagal balance, and in several studies, its increase has been associated with a poor prognosis in both cardiovascular and non-cardiovascular diseases [30,31].

Cardiac ANS causes the release of NE at nerve terminals, which binds to adrenergic receptors and, for 80%, is reuptaken by the fiber endings. However, a smaller fraction of the released NE spills over into the plasma. The regional NE spillover can be estimated and used as a clinical indicator of SNS activity [32]. It has been demonstrated that, in HF, cardiac NE spillover is much higher compared to that measured in other organs (i.e., kidneys and lungs). Cardiac NE spillover is an independent and strong predictor of adverse outcomes in patients with HF [33], and also renal NE spillover is a predictor of mortality in chronic HF [34]. Moreover, cardiac NE spillover has been shown to correlate with the noninvasive HRV measurement low-frequency/high-frequency ratio during sympathetic stimulation in patients with HF [35].

A noninvasive means for measuring the SNS activity is obtained by recording the postganglionic sympathetic nerve activity in skeletal muscle and skin areas (microneurographic technique). Since its inception over 50 years ago, microneurography has been successfully used in HF and other conditions, showing that muscle sympathetic nerve activity (MSNA) is increased in patients with HF and correlates with circulating NE levels [36,37]. Other studies have shown that MSNA is correlated with functional indicators in HF, such as stroke volume [38], and that it might have an independent predictive value on mortality in these patients [39].

A method to study in vivo cardiac SNS dysfunction is by using imaging techniques, either with single-photon emission tomography (SPECT) radioligands such as 123I-MIBG or with positron emission tomography (PET) tracers like 11C-HED. Both compounds are NE analogs that bind to the NE transporter, with PET imaging having a higher spatial resolution compared to SPECT imaging. Several studies in HF have demonstrated that a reduced 123I-mIBG uptake, measured by the lower heart-to-mediastinum ratio (H/M) or increased myocardial 123I-mIBG washout rate, is a marker of abnormal myocardial sympathetic innervation [40,41]. Similarly, a reduced 11C-HED uptake during PET imaging represents cardiac sympathetic denervation. In HF, imaging studies have shown that low 123I-mIBG H/M is an independent predictor of mortality in patients with HF [42,43]. Moreover, regional sympathetic denervation measured by a reduced 11C-HED uptake is a marker of contractile dysfunction and fibrosis in HF patients [44].

Recently, other noninvasive tools have been employed to assess the ANS dysfunction in HF, with one of them being the pupil light reflex (PLR). The pupil radius is controlled by both the parasympathetic and the sympathetic nervous system in response to light exposure, with a mechanism called PLR. The integrity of both ANS branches is essential for a normal PLR response. In patients with HF, PLR is an independent predictor of mortality and readmission due to HF, adding valuable prognostic information [45].

The techniques currently employed in the evaluation of the adrenergic derangement of patients with HF are burdened by poor performance or intrinsic limits of the method that hinders their diffusion and large-scale use, confining them to research protocols or a few specific clinical conditions. The main criticalities are the poor ability to discriminate against the sinoatrial response of sympathetic/vagal stimulation, the nonspecificity for cardiac sympathetic modulation, the costs of machinery and tracers, and the exposure of patients to radionuclides. Accordingly, on top of the above-mentioned tools to noninvasively assess SNS function in HF patients, an expanding field in this area is the one related to the evaluation of blood biomarkers.

## 4. Biomarkers of HF

HF represents a complex clinical process which results in symptoms and signs that can be misleading, as they may be potentially attributable to multiple concomitant conditions, especially in specific populations such as the elderly. These peculiarities often concur with a delayed diagnosis and postponement of appropriate treatment, thus leading to poorer clinical outcomes with high hospitalization rates and costs [46]. In this context, an integrated approach involving biochemical markers may also provide complementary information to allow risk stratification [47]. Accordingly, concerning the symptomatology and clinical objectivity, several biological molecules have the potential to forestall the detection of morphological and functional alterations caused by pathophysiological mechanisms [48]. Indeed, as mentioned above, neurohormonal signaling cascades and pathological remodeling processes anticipate the occurrence of symptomatic ventricular dysfunction [49].

Over the decades, several biomarkers have been proposed in the management of HF, some of which fulfill the criteria for ideal markers to be accurately, quickly, and reproducibly evaluated, not invasively measured, and cheap to assess, with high sensitivity and specificity. They can be categorized into different classes according to the corresponding physiopathology (myocardial wall stress, fibrosis pathway, ANS hyperactivation, and comorbidities-related) or main clinical use (diagnosis and prognosis) [48] (Table 1).

Natriuretic peptides (NP) represent the most extensively employed HF biomarkers, with an established gold standard role in diagnosis and risk stratification [5]. They represent a group of molecules released in response to myocardial stretch, whit-relevant effects on natriuresis, vasodilatation, and fibrosis inhibition [50], which have recently been exploited for new therapeutic approaches [51]. Circulating levels of the brain natriuretic peptide (BNP) and N-Terminal-pro-BNP (NT-pro-BNP), a fragment of the BNP precursor, reflect the NYHA functional class and show an independent linear relationship with in-hospital mortality in patients admitted for both decompensated HFrEF and HFpEF [49,52]. Notably, from a literature review, a 35% augmented risk of death for every 100 pg/mL of BNP increase emerged [53], with a worse prognosis in those experiencing a <50% reduction in NT-pro-BNP levels [54]. Furthermore, NP levels were revealed to be useful in monitoring the effects of treatments with β-blockers [55], ACE-Is/sartans [56], MRAs [57], and cardiac resynchronization therapy [58]. Atrial NP (ANP), released following a stretch of the atrial wall, exerts a similar role of BNP in natriuresis and reduction of the sympathetic tone, but it is burdened by a rapid clearance that limits its routine use [46]. The precursor hormone Mid-Regional pro-ANP (MR-pro-ANP) is more stable, and it has been shown, in the BACH (Biomarkers in Acute Heart Failure) trial, to be comparable to BNP as a diagnostic marker of HF, with an additive prognostic value [59]. It is worth mentioning that NPs are not reliable in all patients, especially in those with multiple comorbidities. BNP concentrations physiologically raise in the elderly [60], and NT-pro-BNP levels are relatively lower in overweight patients [61]. Importantly, the BNP interpretation appears controversial in patients undergoing medical therapy with sacubitril/valsartan, due to its therapeutic mechanism of NP degradation inhibition [62].

Cardiac troponin levels represent another stress/injury biomarker with relevant prognostic implications in acute HF patients [63]. Accordingly, ESC guidelines recommend their measurement in clinical practice to exclude myocardial infarction as a cause of acute HF and for prognostic assessment [5], taking into account that the high-sensitivity cardiac troponin (hs-cTn) peak and trend were associated with 180-day cardiovascular mortality in an acute HF cohort from the RELAX-AHF study [64]. Hs-cTn has also been employed in a stable chronic HF setting; the detectable levels were related to an increased risk of poor clinical outcomes [65].

Galactin-3 is a macrophage-derived mediator related to fibroblast proliferation and activation recently introduced as a biomarker of cardiac remodeling and inflammation [66]. It is related to left ventricle dysfunction and enhanced filling pressures [67,68], and its involvement in HF seems to occur since the early stages of HF, anticipating the onset of symptoms [69]. Otherwise, more than in the diagnostic algorithm, the assessment of the galectin-3 concentration shows a strong prognostic value, as high levels are associated with a worsening of the clinical condition and higher mortality, even in HFpEF [70]. In HFrEF elderly patients, circulating levels of galectin-3 have also been independently associated with frailty [71]. The galectin-3 levels are correlated with inflammatory markers, such as C-reactive protein, vascular endothelial growth factor, and interleukin 6, also being produced in relation to other inflammatory conditions such as obesity [72], which make their interpretation in the perspective of HF insidious, thus limiting the ascertained employment in the clinical routine [73].

Concerning neurohormonal hyperactivation biomarkers, higher circulating levels of adrenomedullin and, specifically, of its mid-regional pro-peptide precursor (MR-pro-ADM), have been detected in patients suffering from chronic HF, diastolic dysfunction, and an increased volume overload [47]. MR-pro-ADM has been shown to represent an independent predictor of HF diagnosis and one-four-year prognosis, independently from NT-pro-BNP [74]. Although promising for prognostic power, adrenomedullin is burdened by a rapid clearance and short half-life, and further data are needed to consider MR-pro-ADM for clinical use, in terms of both the biological activity and cardiac specificity [49].

The Soluble Suppression of Tumorigenicity-2 (ST2) constitutes another inflammatory marker deriving from the interleukin 1-receptor family, which is released in response to the myocardial stretch [75]. ST2 levels are elevated in acute and chronic HF, thus reflecting profibrotic structural heart changes, especially in patients with HFpEF, and correlating to a worse prognosis and higher hospitalization and mortality risks [76,77]. Incremental information regarding the one-year mortality risk derives from its clinical use when associated with the NT-pro-BNP assessment, due to the combination of two relevant different pathways involved in HF pathophysiology [78]. Although being apparently less dependent from other frequent HF comorbidities, such as chronic kidney disease [79], even in dialysis patients [80], ST2 shares with galectin-3 the same limit to also being released in other inflammatory states, so it has not yet been assigned a defined role in clinical practice [73].

Neutrophil gelatinase-associated lipocalin (NGAL) is an acute-phase protein produced by neutrophils and endothelial cells, involved in the response to renal injury [47]. Its role in cardiac function is still unknown, but NGAL values are also high in HF even in the presence of a normal/minimal impaired renal function, and they have been shown to constitute diagnostic and prognostic biomarkers of HF [46]. Among other comorbidities related to HF, it is worth mentioning iron deficiency, a very frequent condition in patients with HFrEF associated with high mortality and morbidity [81], as further confirmed by the evidence of reduced hospitalization rates and improved symptoms after integrative intravenous therapy [82]. Over the last decades, a great stir in the scientific community has focused on the field of circulating microRNAs, which seem to exert a role in HF-related cardiac hypertrophy, cardiomyocyte cell apoptosis, and myocardial fibrosis [83]. In particular, miR423-5p is a potential HF biomarker, but further evidence will be attended to in the next years to effectively validate their use in clinical practice [84].

### Biomarkers of Sympathetic Nervous Activity

Alongside the biomarkers reflecting the pathophysiological processes of myocardial stress, systemic inflammation, and cardiac fibrosis, other molecules are specifically implicated in ANS activation and may potentially be employed in the management of HF patients [26]. A wide array of molecules is implicated in the pathophysiology of HF-related ANS derangement, and in the last decades, a relevant piece of scientific literature focused on them. In the view of an integrated approach, combining the above-mentioned imaging/instrumental tools with novel biomarkers into the management of HF patients, we intend to offer, in this section, a specific focus on laboratory biomarkers of sympathetic overdrive that may potentially support the clinical practice [26].

Circulating levels of plasma CAs, such as their urinary excretion, are higher in HF patients as a consequence of sympathetic overdrive. As mentioned above, higher NE levels determine the activation of α- and β-ARs, increased heart rate, enhanced myocardial contraction, and augmented peripheral vasoconstriction, with pivotal effects on the cardiovascular metabolic demands and energy consumption. For several decades, it has been clear that NE levels are independently related to the mortality of patients with HF [85]. Furthermore, their trends positively correlate with the HF severity, as emerged from the Valsartan Heart Failure Trial (Val-HeFT) [86]. Otherwise, conflicting results emerged from the literature review in terms of the reduction of circulating NE levels through HF gold standard therapies, questioning the actual clinical utility of introducing CA dosing in monitoring patients with HF [87]. Definitely, given the complexity of the techniques for the assay of NE, which require high-performance liquid chromatography [88], the perspective of the routine use of circulating CAs as a HF biomarker is very limited.

Another sympathetic agent released from cardiac fibers, along with CAs, is neuropeptide Y (NPY), a well-known transmitter stimulator of food intake, which also exerts modulatory effects on the cardiovascular system and coronary microvasculature through the strengthening of angiotensin II activity, arteries and veins constriction, attenuation of the parasympathetic tone, promotion of angiogenesis, and cardiac remodeling [26]. The levels of the peptide are elevated in HF patients, and notably, a shred of recent evidence reported higher NPY concentrations in the coronary sinuses of patients undergoing cardiac resynchronization therapy (CRT) to be associated with the composite cardiovascular endpoint (mortality, heart transplant, and ventricular assist device), irrespective of the CRT response [89]. The main criticality in the use of NPY derives from its non-cardiac specificity, predominantly deriving from the hepatic circulation and resulting in becoming less sensitive than NE in quantifying ANS [90].

In the context of the transmitters of the ANS, galanin is another potential biomarker implicated in sympathovagal crosstalk, contributing to the paracrine attenuation of the cardiac cholinergic tone following adrenergic hyperactivity: cardiac parasympathetic fibers express galanin and NPY receptors, whose prolonged activation in HF reduces the acetylcholine release, which, in turn, attenuates the cholinergic neurotransmission in the heart [91,92]. In addition to experimental models, in recent years, the relationship of galanin with other biomarkers in populations of patients affected by HF has been highlighted, but to date, its role has not yet been adequately defined [93].

Chromogranin A exerts a relevant role in modulating the adrenergic system, and its blood levels are higher in HF patients, relating to negative outcomes [94]. It is the precursor of catestatin (CST), produced by neuroendocrine tissues and nerve fibers, which exerts the negative regulation of CA release through the neuronal nicotinic acetylcholine receptor (nAchR) [95]. The pathophysiological role of CTS on the cardiovascular system is realized through reduced arterial blood pressure, reduced release of NPY and ATP beyond CAs, decreased inflammation and thrombogenicity, diminished ventricular remodeling. Further, recent evidence allows to speculate on a plausible role as an indirect marker of ANS activity [26]. Indeed, the analysis from the CATSTAT-HF study reported CTS values to be higher in acute HF patients and to correlate with both the NYHA functional classes and ischemic etiology, independently from the LVEF phenotypes [96]. However, the evidence is still too limited, as the effect of therapeutic agents on the CTS levels should be clarified.

## 5. Lymphocyte GRK2 as Biomarkers of HF

More than three decades of investigations support the key pathogenic role of cardiac GRK2 levels in HF onset and progression [9], and preclinical studies have shown that cardiac GRK2 levels depend on the status of the cardiac β-AR system; indeed, they are increased when the pathway is hyperactivated and reduced if the receptors are blocked [97]. However, studies in transgenic mice have shown that over a 200-fold cardiac overexpression of β1AR ultimately determines the myocardial dysfunction, following the short-term improvement of the heart function [98]. Furthermore, the effect of β2AR overexpression depends on the levels of the transgene. Indeed, up to 60-fold β2AR basal levels result in enhanced cardiac function, whereas a higher expression is responsible for HF and dilated cardiomyopathy development [99]. Sustained ANS hyperactivity determines the enhanced cardiac GRK2 expression, which, in turn, results in β-AR downregulation/desensitization. Besides the relevant therapeutic implications of GRK2 inhibition in HF, the cardiac GRK2 expression may provide relevant information regarding post-MI cardiac remodeling and HF progression. Indeed, the GRK2 protein levels, more closely reflecting the sustained hyperactivation of β-AR by CAs, may represent a more stable surrogate of ANS hyperactivity than the circulating NE levels, adding important information to the cardiac β-AR function. Notably, a study by Iaccarino and collaborators demonstrated that the GRK2 levels, measured in HF patients’ peripheral lymphocytes, mirror the kinase expression in the myocardium, reflecting the loss of β-AR responsiveness, the degree of cardiac dysfunction, and the severity of the syndrome. Moreover, the authors reported a direct correlation between the lymphocyte GRK2 levels and peripheral NE circulating levels and an inverse correlation with the cardiac β-AR function in patients with HF. This study paved the way for a potential clinical application of lymphocyte GRK2 levels, allowing to speculate on white blood cells as a surrogate of cardiac GRK2 in HF patients [100].

ANS hyperactivity is a systemic phenomenon, and an increased GRK2 expression has been reported in several cell types and tissues, including the brain, vessels, and adrenal glands, in experimental models of diseases characterized by adrenergic derangement. Thus, both the failing heart and peripheral lymphocytes are exposed to a similar milieu characterized by increased CAs, which can sustainably activate β-ARs of white blood cells, thus triggering GRK2 upregulation. In patients with advanced end-stage HF undergoing left ventricular unloading, a similar reduction in the cardiac and lymphocyte GRK2 levels was observed, suggesting the potentiality of lymphocyte GRK2 at predicting the patient responsiveness to specific therapies [101]. Moreover, this latter finding has been confirmed and also extended to patients undergoing continuous-flow left ventricle unloading [102]. In HF patients undergoing structured rehabilitation programs, exercise-induced reduction in the lymphocyte GRK2 expression was associated with a better prognosis, while a poor outcome was reported in patients with no evidence of training-induced changes in the blood GRK2 levels [103]. Further data have been obtained from patients undergoing cardiac transplantation: a significant decrease in blood GRK2 has been observed consistently with the improved contractility of the transplanted heart [104].

The prognostic potentialities of blood GRK2 levels have been also explored in a larger HF population, showing lymphocyte kinase expression to significantly predict mortality with additional independent prognostic information to those derived from well-established predictors, such as the LVEF and NT-pro-BNP serum levels [105]. Taking together, the above-mentioned studies support the role of white cell GRK2 to serve as a biomarker in patients with chronic HF, to both guide specific therapies and predict outcomes. Moreover, GRK2 has the potentiality to add information, over the currently available biomarkers, on HF-related ANS hyperactivity and on its implication in the cardiac β-AR signaling function, which owns a crucial role in HF pathophysiology. White cell GRK2 levels have been reported to also increase in acute ST segment elevation in myocardial infarction (MI) patients and to predict post-MI cardiac remodeling [106].

In clinical practice, blood biomarkers generally claim indisputable advantages in terms of time and cost-effectiveness; furthermore, they favor the compliance of patients who prefer to undergo a blood sample rather than instrumental/invasive techniques that are sometimes laborious. It is worth mentioning that the application of blood GRK2 as a HF biomarker in clinical practice is burdened by some limitations. First, the isolation of mononuclear cells by the ficoll gradient on fresh blood is needed, since GRK2 cannot be detected in circulating blood. Moreover, the methodology adopted to assess the GRK2 levels in most studies was Western blotting, a semiquantitative technique. This implies that the results obtained from a diverse population and different laboratories are not comparable. Thus, the development and validation of a high-throughput assay for GRK2 detection, such as ELISA, are needed to translate this proof-of-concept into clinical practice.

## 6. Future Perspectives

Despite the high prevalence of the syndrome and the non-negligible arsenal of pharmacological strategies tested and approved in the recent years, HF is still burdened by the lack of ideal biomarkers, able to represent all the pathophysiological pathways overcoming the methodological limits that characterize the currently available molecules. In this context, waiting for further evidence, a multi-markers approach appears indispensable, combining several pieces of information related to different aspects of HF pathophysiology in order to improve the management and risk stratification of affected patients. This is particularly true about the hyperactivity of ANS, for which integration with direct and indirect instrumental tools assessing the sympathetic nervous system appears essential. Aiming at identifying the best techniques and the most appropriate approaches for a comprehensive assessment of ANS function in HF, it would be desirable to combine the data deriving from centers with greater experience in the study of the sympathetic derangement in HF in order to integrate the results of several assessment tools and possibly propose a holistic diagnostic algorithm that may overcome the limits of each single component.

Previous studies have indicated peripheral blood cells as a surrogate of the alterations in cardiac β-AR signaling occurring in HF. Despite the advantages provided by the blood molecule assay over more elaborate/invasive tools, and the evidence supporting lymphocytic GRK2 detection as an implemental informative technique for ANS assessment, the above-mentioned methodological limitations have slowed down its introduction in clinical practice as a potential biomarker of sympathetic nervous system activity. Further studies will be needed to better define the potentiality and feasibility of blood GRK2 to serve as a biomarker providing information not only on the ANS status but, also, on several pathways frequently altered in HF patients (i.e., insulin signaling) [20].

Nonetheless, the exceptional technological advances applied to medicine in the last decade allow us to envisage future scenarios for the development of HF biomarkers [47]. In particular, metabolomics will allow the complete evaluation of the metabolic byproducts in order to identify the metabolic signature profiles in particular populations, which may constitute diagnostic and prognostic tools for the HF population. Similarly, the comprehensive study of RNA transcripts produced in specific circumstances might enable the identification of different gene expressions in particular cell types or as pharmacological effects. In the perspective of the personalization of the care era, genetic testing may exert a pivotal role in clarifying any genetic cause of disease or in monitoring treatment approaches.

## 7. Conclusive Remarks

HF is characterized by a decreased myocardial pump function and enhanced pressure load, with a tremendous burden on global healthcare systems. Several biomarkers have been identified in order to support clinical and instrumental testing in both the diagnostic process and prognostic assessment of such a complex systemic syndrome. One of the pillars of HF is represented by sympathetic nervous system hyperactivation, which importantly impacts patients’ outcomes. To date, the assessment of ANS activity in HF is challenging due to the intrinsic features of both the proposed instrumental tools and tested biomarkers, that limit the exploration of this pathological mechanism in the affected patient. Given the ability to reflect the myocardial β-AR signaling pathway, the evaluation of the lymphocyte GRK2 levels has been shown to provide prognostic information related to sympathetic overdrive additional and independent from other biomarkers. Further evidence will be needed to ascertain the effectiveness of adrenergic biomarkers in HF prognostic stratification and to develop other tools for the implementation of an ANS assessment in routine clinical practice.

## Figures and Tables

**Table 1:** Summary of the main HF biomarkers.

Biomarkers	Cut Off Values	Production	Increasing in	Hf Phenotype	Role
**BNP**	>35 pg/mL	released from myocytes under stress	HF, Aging, LVH, CKD, AS, MI, AF, Obesity	HFrEF > HFpEF	Diagnosis, Prognosis, Follow up
**NT PRO BNP**	>125 pg/mL	fragment of BNP precursor	HF, Aging, LVH, CKD, AS, MI, AF	HFrEF > HFpEF	Diagnosis, Prognosis, Follow up
**MR PRO ANP**	>127 pmol/L	atrial wall as result of stretch	HF, AS, Sepsis, MI, AF, Burns	HFrEF > HFpEF	Diagnosis, Prognosis, Follow up
**HS-CIN TROPONIN**	>34.2 pg/mL	cardiomyocytes injury	HF, MI, Myocarditis, CKD, Sepsis, Hypothyroidism, Trauma	HFrEF > HFpEF	Diagnosis, Prognosis
**GALECTIN 3**	<17.8 ng/mL	fibroblast proliferation and activation	HF, Aging, DM, CKD, IPF, Obesity, Cirrhosis, Cancer, Inflammatory states	HFpEF > HFrEF	Prognosis
**MR-PROADM**	0.10–0.64 nmol/L	released in several tissue as result of increased pressure and volume overload	HF, MI, CAD, Hypertension, CKD, Sepsis, Cancer	HFmrEF	Prognosis
**ST2**	>30 ng/mL	myocardial stretch	HF, CAD, IS	HFpEF > HFrEF	Prognosis
**NGAL**	50 ng/mL	neutrophils and endothelial cells, involved in response renal injury	HF, RI	HFrEF > HFpEf	Diagnosis, Prognosis
**IRON DEFICIENCY**	Ferritin <15 µg/L	multifactorial condition	HF, IDA, IM, Bleeding	HFrEF > HFpEF	Prognosis
**NE**	>480 pg/dl	Neuroendocrine cells as result of sympathetic overdrive	HF, MI, Hypertension, Pheochromocytoma, Cushing, Stress	HFrEF>HFpEF	Prognosis
**NPY**	>130 pg/mL	Neuroendocrine cells as result of sympathetic overdrive	HF, Obesity, Stress	HFrEF	Prognosis
**GALANIN**	To be determined yet	Neuroendocrine and gastrointestinal cells	HF, Hypertension, Pain, SL, Cancer	HFpEF	To be further elucidated
**CHROMOGRANIN A** **/CST**	>19.73 ng/mL	Neuroendocrine and myocardial cells	HF, CAD, Sepsis	Independent from LVEF	Prognosis

AF: atrial fibrillation, AS: aortic stenosis, BNP: brain natriuretic peptide, CAD: coronary artery disease, CKD: chronic kidney disease, CST: catestatin, DM: diabetes mellitus, HF: heart failure, HFmrEF: Heart Failure with Mid-Range Ejection Fraction, HFpEF: Heart Failure with Preserved Ejection Fraction, HFrEF: Heart Failure with Reduced Ejection Fraction, HS-CIN: High-Sensitivity Cardiac Troponin, IDA: iron deficiency anemia, IM: intestinal malabsorption, IPF: idiopathic pulmonary fibrosis, IS: inflammatory states, LVEF: left ventricular ejection fraction, LVH: left ventricular hypertrophy, MI: myocardial infarction, NE: norepinephrine, NGAL: neutrophil gelatinase-associated lipocalin, NPY: neuropeptide Y, PAH: pulmonary arterial hypertension, RI: renal injury, SL: sleep regulation, and ST2: Soluble Suppression of Tumorigenicity.

## Data Availability

Not applicable.

## References

[1] Savarese G., Lund L.H. (2017). Global Public Health Burden of Heart Failure. Card. Fail. Rev..

[2] Lippi G., Sanchis-Gomar F. (2020). Global epidemiology and future trends of heart failure. AME Med. J..

[3] Dharmarajan K., Rich M.W. (2017). Epidemiology, Pathophysiology, and Prognosis of Heart Failure in Older Adults. Hear. Fail. Clin..

[4] De Lucia C., Eguchi A., Koch W.J. (2018). New Insights in Cardiac β-Adrenergic Signaling During Heart Failure and Aging. Front. Pharmacol..

[5] Ponikowski P., Voors A.A., Anker S.D., Bueno H., (Uk) J.G.F.C., (Uk) A.J.S.C., Falk V., González-Juanatey J.R., Harjola V.-P., Jankowska E.A. (2016). 2016 ESC Guidelines for the diagnosis and treatment of acute and chronic heart failure. Eur. J. Hear. Fail..

[6] Bencivenga L., Liccardo D., Napolitano C., Visaggi L., Rengo G., Leosco D. (2019). β-Adrenergic receptor signaling and heart failure: From bench to bedside. Heart Fail. Clin..

[7] Triposkiadis F., Karayannis G., Giamouzis G., Skoularigis J., Louridas G., Butler J. (2009). The Sympathetic Nervous System in Heart Failure. J. Am. Coll. Cardiol..

[8] Santulli G., Iaccarino G. (2016). Adrenergic signaling in heart failure and cardiovascular aging. Maturitas.

[9] Cannavo A., Komici K., Bencivenga L., D’Amico M.L., Gambino G., Liccardo D., Ferrara N., Rengo G. (2018). GRK2 as a therapeutic target for heart failure. Expert Opin. Ther. Targets.

[10] Florea V.G., Cohn J.N. (2014). The Autonomic Nervous System and Heart Failure. Circ. Res..

[11] Lymperopoulos A., Rengo G., Koch W.J. (2007). Adrenal adrenoceptors in heart failure: Fine-tuning cardiac stimulation. Trends Mol. Med..

[12] Moniotte S., Kobzik L., Feron O., Trochu J.-N., Gauthier C., Balligand J.-L. (2001). Upregulation of β 3 -Adrenoceptors and Altered Contractile Response to Inotropic Amines in Human Failing Myocardium. Circulation.

[13] Myagmar B.-E., Flynn J.M., Cowley P.M., Swigart P.M., Montgomery M.D., Thai K., Nair D.R., Gupta R., Deng D.X., Hosoda C. (2017). Adrenergic Receptors in Individual Ventricular Myocytes. Circ. Res..

[14] Gauthier C., Leblais V., Kobzik L., Trochu J.N., Khandoudi N., Bril A., Balligand J.-L., Le Marec H. (1998). The negative inotropic effect of beta3-adrenoceptor stimulation is mediated by activation of a nitric oxide synthase pathway in human ventricle. J. Clin. Investig..

[15] Lefkowitz R.J. (2005). Transduction of Receptor Signals by -Arrestins. Science.

[16] Lymperopoulos A., Rengo G., Koch W.J. (2013). Adrenergic Nervous System in Heart Failure. Circ. Res..

[17] Woodall M.C., Ciccarelli M., Woodall B.P., Koch W.J. (2014). G protein-coupled receptor kinase 2: A link between myocardial contractile function and cardiac metabolism. Circ. Res..

[18] Woodall B.P., Gresham K.S., Woodall M.A., Valenti M.-C., Cannavo A., Pfleger J., Chuprun J.K., Drosatos K., Koch W.J. (2019). Alteration of myocardial GRK2 produces a global metabolic phenotype. JCI Insight.

[19] Cannavo A., Marzano F., Elia A., Liccardo D., Bencivenga L., Gambino G., Perna C., Rapacciuolo A., Cittadini A., Ferrara N. (2019). Aldosterone Jeopardizes Myocardial Insulin and β-Adrenergic Receptor Signaling via G Protein-Coupled Receptor Kinase 2. Front. Pharmacol..

[20] Murga C., Arcones A.C., Cruces-Sande M., Briones A.M., Salaices M., Mayor F. (2019). G Protein-Coupled Receptor Kinase 2 (GRK2) as a Potential Therapeutic Target in Cardiovascular and Metabolic Diseases. Front. Pharmacol..

[21] Thireau J., Karam S., Roberge S., Roussel J., Aimond F., Cassan C., Gac A., Babuty D., Le Guennec J.-Y., Lacampagne A. (2014). β-Adrenergic blockade combined with subcutaneous B-type natriuretic peptide: A promising approach to reduce ventricular arrhythmia in heart failure?. Heart.

[22] Cipolletta E., Campanile A., Santulli G., Sanzari E., Leosco D., Campiglia P., Trimarco B., Iaccarino G. (2009). The G protein coupled receptor kinase 2 plays an essential role in beta-adrenergic receptor-induced insulin resistance. Cardiovasc. Res..

[23] Watari K., Nakaya M., Kurose H. (2014). Multiple functions of G protein-coupled receptor kinases. J. Mol. Signal..

[24] Orso F., Baldasseroni S., Maggioni A.P. (2009). Heart Rate in Coronary Syndromes and Heart Failure. Prog. Cardiovasc. Dis..

[25] Fox K., Ford I., Steg P.G., Tendera M., Ferrari R. (2008). Ivabradine for patients with stable coronary artery disease and left-ventricular systolic dysfunction (BEAUTIFUL): A randomised, double-blind, placebo-controlled trial. Lancet.

[26] Borovac J.A., D’Amario D., Bozic J., Glavas D. (2020). Sympathetic nervous system activation and heart failure: Current state of evidence and the pathophysiology in the light of novel biomarkers. World J. Cardiol..

[27] Ewing D.J., Martyn C.N., Young R.J., Clarke B.F. (1985). The Value of Cardiovascular Autonomic Function Tests: 10 Years Experience in Diabetes. Diabetes Care.

[28] Patel H., Ozdemir B.A., Patel M., Xiao H.B., Poole-Wilson P.A., Rosen S.D. (2011). Impairment of autonomic reactivity is a feature of heart failure whether or not the left ventricular ejection fraction is normal. Int. J. Cardiol..

[29] Shaffer F., Ginsberg J.P. (2017). An Overview of Heart Rate Variability Metrics and Norms. Front. Public Health.

[30] Patel V.N., Pierce B.R., Bodapati R.K., Brown D.L., Ives D.G., Stein P.K. (2017). Association of holter-derived heart rate variability parameters with the development of congestive heart failure in the cardiovascular health study. JACC Hear. Fail..

[31] Femminella G.D., Rengo G., Komici K., Iacotucci P., Petraglia L., Pagano G., De Lucia C., Canonico V., Bonaduce D., Leosco D. (2014). Autonomic Dysfunction in Alzheimer’s Disease: Tools for Assessment and Review of the Literature. J. Alzheimer’s Dis..

[32] Hasking G.J., Esler M.D., Jennings G.L., Burton D., Johns J.A., Korner P.I. (1986). Norepinephrine spillover to plasma in patients with congestive heart failure: Evidence of increased overall and cardiorenal sympathetic nervous activity. Circulation.

[33] Kaye D.M., Lefkovits J., Jennings G.L., Bergin P., Broughton A., Esler M.D. (1995). Adverse consequences of high sympathetic nervous activity in the failing human heart. J. Am. Coll. Cardiol..

[34] Petersson M., Friberg P., Eisenhofer G., Lambert G., Rundqvist B. (2005). Long-term outcome in relation to renal sympathetic activity in patients with chronic heart failure. Eur. Hear. J..

[35] Tygesen H., Rundqvist B., Waagstein F., Wennerblom B. (2001). Heart rate variability measurement correlates with cardiac norepinephrine spillover in congestive heart failure. Am. J. Cardiol..

[36] Grassi G., Colombo M., Seravalle G., Spaziani D., Mancia G. (1998). Dissociation Between Muscle and Skin Sympathetic Nerve Activity in Essential Hypertension, Obesity, and Congestive Heart Failure. Hypertension.

[37] Leimbach W.N., Wallin B.G., Victor R.G., Aylward P.E., Sundlöf G., Mark A.L. (1986). Direct evidence from intraneural recordings for increased central sympathetic outflow in patients with heart failure. Circulation.

[38] Ferguson D.W., Berg W.J., Sanders J.S., Kempf J.S. (1990). Clinical and hemodynamic correlates of sympathetic nerve activity in normal humans and patients with heart failure: Evidence from direct micronenrographic recordings. J. Am. Coll. Cardiol..

[39] Barretto A.C., Santos A.C., Munhoz R., Rondon M.U., Franco F.G., Trombetta I.C., Roveda F., De Matos L.N., Braga A.M., Middlekauff H.R. (2009). Increased muscle sympathetic nerve activity predicts mortality in heart failure patients. Int. J. Cardiol..

[40] Jacobson A.F., Senior R., Cerqueira M.D., Wong N.D., Thomas G.S., Lopez V.A., Agostini D., Weiland F., Chandna H., Narula J. (2010). Myocardial Iodine-123 Meta-Iodobenzylguanidine Imaging and Cardiac Events in Heart Failure. J. Am. Coll. Cardiol..

[41] Komici K., Bencivenga L., Paolillo S., Gargiulo P., Formisano R., Assante R., Nappi C., Marsico F., D’Antonio A., De Simini G. (2019). Impact of body mass index on cardiac adrenergic derangement in heart failure patients: A 123I-mIBG imaging study. Eur. J. Nucl. Med. Mol. Imaging.

[42] Narula J., Gerson M., Thomas G.S., Cerqueira M.D., Jacobson A.F. (2015). 123I-MIBG Imaging for Prediction of Mortality and Potentially Fatal Events in Heart Failure: The ADMIRE-HFX Study. J. Nucl. Med..

[43] Nakata T., Nakajima K., Yamashina S., Yamada T., Momose M., Kasama S., Matsui T., Matsuo S., Travin M.I., Jacobson A.F. (2013). A Pooled Analysis of Multicenter Cohort Studies of 123I-mIBG Imaging of Sympathetic Innervation for Assessment of Long-Term Prognosis in Heart Failure. JACC: Cardiovasc. Imaging.

[44] Aikawa T., Naya M., Obara M., Oyama-Manabe N., Manabe O., Magota K., Ito Y.M., Katoh C., Tamaki N. (2017). Regional interaction between myocardial sympathetic denervation, contractile dysfunction, and fibrosis in heart failure with preserved ejection fraction: 11C-hydroxyephedrine PET study. Eur. J. Nucl. Med. Mol. Imaging.

[45] Nozaki K., Hamazaki N., Yamamoto S., Kamiya K., Tanaka S., Ichikawa T., Nakamura T., Yamashita M., Maekawa E., Matsunaga A. (2020). Prognostic value of pupil area for all-cause mortality in patients with heart failure. ESC Hear. Fail..

[46] Nadar S.K., Shaikh M.M. (2019). Biomarkers in Routine Heart Failure Clinical Care. Card. Fail. Rev..

[47] Ibrahim N.E., Januzzi J.L. (2018). Established and Emerging Roles of Biomarkers in Heart Failure. Circ. Res..

[48] Altay H. (2018). Biomarkers and Heart Failure. In Biomarker - Indicator of Abnormal Physiological Process.

[49] Gaggin H.K., Januzzi J.L. (2013). Biomarkers and diagnostics in heart failure. Biochim. et Biophys. Acta (BBA) - Mol. Basis Dis..

[50] Lin D.C., Diamandis E.P., Januzzi J.L., Maisel A., Jaffe A.S., Clerico A. (2014). Natriuretic Peptides in Heart Failure. Clin. Chem..

[51] McMurray J.J., Packer M., Desai A.S., Gong J., Lefkowitz M.P., Rizkala A.R., Rouleau J.L., Shi V.C., Solomon S.D., Swedberg K. (2014). Angiotensin–Neprilysin Inhibition versus Enalapril in Heart Failure. N. Engl. J. Med..

[52] Fonarow G.C., Peacock W.F., Phillips C.O., Givertz M.M., Lopatin M. (2007). Admission B-Type Natriuretic Peptide Levels and In-Hospital Mortality in Acute Decompensated Heart Failure. J. Am. Coll. Cardiol..

[53] Doust J.A., Pietrzak E., Dobson A., Glasziou P. (2005). How well does B-type natriuretic peptide predict death and cardiac events in patients with heart failure: Systematic review. BMJ.

[54] Michtalik H.J., Yeh H.-C., Campbell C.Y., Haq N., Park H., Clarke W., Brotman D.J. (2011). Acute Changes in N-Terminal Pro-B-Type Natriuretic Peptide During Hospitalization and Risk of Readmission and Mortality in Patients With Heart Failure. Am. J. Cardiol..

[55] Stanek B., Frey B., Hülsmann M., Berger R., Sturm B., Strametz-Juranek J., Bergler-Klein J., Moser P., Bojic A., Hartter E. (2001). Prognostic evaluation of neurohumoral plasma levels before and during beta-blocker therapy in advanced left ventricular dysfunction. J. Am. Coll. Cardiol..

[56] Yoshimura M., Mizuno Y., Nakayama M., Sakamoto T., Sugiyama S., Kawano H., Soejima H., Hirai N., Saito Y., Nakao K. (2002). B-type natriuretic peptide as a marker of the effects of enalapril in patients with heart failure. Am. J. Med..

[57] Berry C., Murphy N., De Vito G., Galloway S., Seed A., Fisher C., Sattar N., Vallance P., Hillis W.S., McMurray J. (2007). Effects of aldosterone receptor blockade in patients with mild-moderate heart failure taking a beta-blocker. Eur. J. Hear. Fail..

[58] Menardi E., Vado A., Rossetti G., Racca E., Conte E., Deorsola A., Bobbio M., Feola M. (2008). Cardiac Resynchronization Therapy Modifies the Neurohormonal Profile, Hemodynamic and Functional Capacity in Heart Failure Patients. Arch. Med Res..

[59] Maisel A., Mueller C., Nowak R., Peacock W.F., Landsberg J.W., Ponikowski P., Mockel M., Hogan C., Wu A.H.B., Richards M. (2010). Mid-Region Pro-Hormone Markers for Diagnosis and Prognosis in Acute Dyspnea. J. Am. Coll. Cardiol..

[60] Kim H.-N., Januzzi J.L. (2011). Natriuretic Peptide Testing in Heart Failure. Circulation.

[61] Bayes-Genis A., Lloyd-Jones D.M., Van Kimmenade R.R.J., Lainchbury J.G., Richards A.M., Ordoñez-Llanos J., Santaló M., Pinto Y.M., Januzzi J.L. (2007). Effect of Body Mass Index on Diagnostic and Prognostic Usefulness of Amino-Terminal Pro–Brain Natriuretic Peptide in Patients With Acute Dyspnea. Arch. Intern. Med..

[62] Voors A., Dorhout B., Van Der Meer P. (2013). The potential role of valsartan + AHU377 (LCZ696) in the treatment of heart failure. Expert Opin. Investig. Drugs.

[63] Peacock W.F., De Marco T., Fonarow G.C., Diercks D.B., Wynne J., Apple F.S., Wu A.H. (2008). Cardiac Troponin and Outcome in Acute Heart Failure. N. Engl. J. Med..

[64] Felker G.M., Mentz R.J., Teerlink J.R., Voors A.A., Pang P.S., Ponikowski P., Greenberg B.H., Filippatos G., Davison B.A., Cotter G. (2015). Serial high sensitivity cardiac troponin T measurement in acute heart failure: Insights from the RELAX-AHF study. Eur. J. Hear. Fail..

[65] Latini R., Masson S., Anand I.S., Missov E., Carlson M., Vago T., Angelici L., Barlera S., Parrinello G., Maggioni A.P. (2007). Prognostic Value of Very Low Plasma Concentrations of Troponin T in Patients With Stable Chronic Heart Failure. Circulation.

[66] McCullough P., Olobatoke A., Olobatoke T.E. (2011). Galectin-3: A novel blood test for the evaluation and management of patients with heart failure. Rev. Cardiovasc. Med..

[67] Michalski B., Trzciński P., Kupczyńska K., Miśkowiec D., Pęczek ę., Nawrot B., Lipiec P., Kasprzak J.D. (2017). The differences in the relationship between diastolic dysfunction, selected biomarkers and collagen turn-over in heart failure patients with preserved and reduced ejection fraction. Cardiol. J..

[68] Lok D.J., Lok S.I., De La Porte P.W.B.-A., Badings E., Lipsic E., Van Wijngaarden J., De Boer R.A., Van Veldhuisen D.J., Van Der Meer P. (2012). Galectin-3 is an independent marker for ventricular remodeling and mortality in patients with chronic heart failure. Clin. Res. Cardiol..

[69] Sharma U.C., Pokharel S., Van Brakel T.J., Van Berlo J.H., Cleutjens J.P.M., Schroen B., Andreé S., Crijns H.J.G.M., Gabius H.-J., Maessen J. (2004). Galectin-3 Marks Activated Macrophages in Failure-Prone Hypertrophied Hearts and Contributes to Cardiac Dysfunction. Circulation.

[70] De Boer R.A., Lok D.J.A., Jaarsma T., Van Der Meer P., Voors A.A., Hillege H.L., Van Veldhuisen D.J. (2010). Predictive value of plasma galectin-3 levels in heart failure with reduced and preserved ejection fraction. Ann. Med..

[71] Komici K., Gnemmi I., Bencivenga L., Vitale D.F., Rengo G., Di Stefano A., Eleuteri E. (2020). Impact of Galectin-3 Circulating Levels on Frailty in Elderly Patients with Systolic Heart Failure. J. Clin. Med..

[72] Martínez-Martínez E., López-Ándres N., Jurado-López R., Rousseau E., Bartolomé M.V., Fernández-Celis A., Rossignol P., Islas F., Antequera A., Prieto S. (2015). Galectin-3 Participates in Cardiovascular Remodeling Associated With Obesity. Hypertension.

[73] Piek A., Du W., De Boer R.A., Silljé H.H.W. (2018). Novel heart failure biomarkers: Why do we fail to exploit their potential?. Crit. Rev. Clin. Lab. Sci..

[74] Shah R.V., Truong Q.A., Gaggin H.K., Pfannkuche J., Hartmann O., Januzzi J.L. (2012). Mid-regional pro-atrial natriuretic peptide and pro-adrenomedullin testing for the diagnostic and prognostic evaluation of patients with acute dyspnoea. Eur. Hear. J..

[75] Sanada S., Hakuno D., Higgins L.J., Schreiter E.R., McKenzie A.N., Lee R.T. (2007). IL-33 and ST2 comprise a critical biomechanically induced and cardioprotective signaling system. J. Clin. Investig..

[76] Mueller T., Dieplinger B., Gegenhuber A., Poelz W., Pacher R., Haltmayer M. (2008). Increased Plasma Concentrations of Soluble ST2 are Predictive for 1-Year Mortality in Patients with Acute Destabilized Heart Failure. Clin. Chem..

[77] Nagy A.I., Hage C., Merkely B., Donal E., Daubert J.-C., Linde C., Lund L.H., Manouras A. (2018). Left atrial rather than left ventricular impaired mechanics are associated with the pro-fibrotic ST2 marker and outcomes in heart failure with preserved ejection fraction. J. Intern. Med..

[78] Januzzi J.L., Peacock W.F., Maisel A.S., Chae C.U., Jesse R.L., Baggish A.L., O’Donoghue M., Sakhuja R., Chen A.A., Van Kimmenade R.R. (2007). Measurement of the Interleukin Family Member ST2 in Patients With Acute Dyspnea. J. Am. Coll. Cardiol..

[79] Bayes-Genis A., Zamora E., De Antonio M., Galán A., Vila J., Urrutia A., Díez C., Coll R., Altimir S., Lupón J. (2013). Soluble ST2 Serum Concentration and Renal Function in Heart Failure. J. Card. Fail..

[80] Homsak E., Ekart R. (2018). ST2 as a novel prognostic marker in end-stage renal disease patients on hemodiafiltration. Clin. Chim. Acta.

[81] Jankowska E.A., Rozentryt P., Witkowska A., Nowak J., Hartmann O., Ponikowska B., Borodulin-Nadzieja L., Banasiak W., Polonski L., Filippatos G. (2010). Iron deficiency: An ominous sign in patients with systolic chronic heart failure. Eur. Hear. J..

[82] Jankowska E.A., Tkaczyszyn M., Suchocki T., Drozd M., Von Haehling S., Doehner W., Banasiak W., Filippatos G., Anker S.D., Ponikowski P. (2016). Effects of intravenous iron therapy in iron-deficient patients with systolic heart failure: A meta-analysis of randomized controlled trials. Eur. J. Hear. Fail..

[83] De Lucia C., Komici K., Borghetti G., Femminella G.D., Bencivenga L., Cannavo A., Corbi G., Ferrara N., Houser S.R., Koch W.J. (2017). microRNA in Cardiovascular Aging and Age-Related Cardiovascular Diseases. Front. Med..

[84] Yan H., Ma F., Zhang Y., Wang C., Qiu D., Zhou K., Hua Y., Li Y. (2017). miRNAs as biomarkers for diagnosis of heart failure. Medicine.

[85] Cohn J.N., Levine T.B., Olivari M.T., Garberg V., Lura D., Francis G.S., Simon A.B., Rector T. (1984). Plasma Norepinephrine as a Guide to Prognosis in Patients with Chronic Congestive Heart Failure. N. Engl. J. Med..

[86] Anand I.S., Fisher L.D., Chiang Y.-T., Latini R., Masson S., Maggioni A.P., Glazer R.D., Tognoni G., Cohn J.N. (2003). Changes in Brain Natriuretic Peptide and Norepinephrine Over Time and Mortality and Morbidity in the Valsartan Heart Failure Trial (Val-HeFT). Circulation.

[87] Givertz M.M., Braunwald E. (2004). Neurohormones in heart failure: Predicting outcomes, optimizing care. Eur. Hear. J..

[88] Hjemdahl P. (1993). Plasma catecholamines—Analytical challenges and physiological limitations. Baillière’s Clin. Endocrinol. Metab..

[89] Ajijola O.A., Chatterjee N.A., Gonzales M.J., Gornbein J., Liu K., Li D., Paterson D.J., Shivkumar K., Singh J.P., Herring N. (2020). Coronary Sinus Neuropeptide Y Levels and Adverse Outcomes in Patients With Stable Chronic Heart Failure. JAMA Cardiol..

[90] Morris M.J., Cox H.S., Lambert G.W., Kaye D.M., Jennings G.L., Meredith I.T., Esler M.D. (1997). Region-Specific Neuropeptide Y Overflows at Rest and During Sympathetic Activation in Humans. Hypertension.

[91] Herring N., Cranley J., Lokale M.N., Li D., Shanks J., Alston E.N., Girard B.M., Carter E., Parsons R.L., Habecker B.A. (2012). The cardiac sympathetic co-transmitter galanin reduces acetylcholine release and vagal bradycardia: Implications for neural control of cardiac excitability. J. Mol. Cell. Cardiol..

[92] Smith-White M., Iismaa T.P., Potter E.K. (2003). Galanin and neuropeptide Y reduce cholinergic transmission in the heart of the anaesthetised mouse. Br. J. Pharmacol..

[93] Gür D. (2017). Özkaramanlı; Savaş, G.; Akyüz, A.; Alpsoy, Şeref Role of sympathetic cotransmitter galanin on autonomic balance in heart failure: An active player or a bystander?. Anatol. J. Cardiol..

[94] Ceconi C., Ferrari R., Bachetti T., Opasich C., Volterrani M., Colombo B., Parrinello G., Corti A. (2002). Chromogranin A in heart failure. A novel neurohumoral factor and a predictor for mortality. Eur. Hear. J..

[95] Mahata S.K., Mahata M., Fung M.M., O’Connor D.T. (2010). Catestatin: A multifunctional peptide from chromogranin A. Regul. Pept..

[96] Borovac J.A., Glavas D., Grabovac Z.S., Domic D.S., D’Amario D., Bozic J. (2019). Catestatin in Acutely Decompensated Heart Failure Patients: Insights from the CATSTAT-HF Study. J. Clin. Med..

[97] Iaccarino G., Tomhave E.D., Lefkowitz R.J., Koch W.J. (1998). Reciprocal In Vivo Regulation of Myocardial G Protein–Coupled Receptor Kinase Expression by β-Adrenergic Receptor Stimulation and Blockade. Circulation.

[98] Engelhardt S., Hein L., Wiesmann F., Lohse M.J. (1999). Progressive hypertrophy and heart failure in 1-adrenergic receptor transgenic mice. Proc. Natl. Acad. Sci. USA.

[99] Liggett S.B., Tepe N.M., Lorenz J.N., Canning A.M., Jantz T.D., Mitarai S., Yatani A., Dorn G.W. (2000). Early and Delayed Consequences of 2-Adrenergic Receptor Overexpression in Mouse Hearts: Critical Role for Expression Level. Circulation.

[100] Iaccarino G., Barbato E., Cipolletta E., De Amicis V., Margulies K.B., Leosco D., Trimarco B., Koch W.J. (2005). Elevated myocardial and lymphocyte GRK2 expression and activity in human heart failure. Eur. Hear. J..

[101] Hata J.A., Williams M.L., Schroder J.N., Lima B., Keys J.R., Blaxall B.C., Petrofski J.A., Jakoi A., Milano C.A., Koch W.J. (2006). Lymphocyte Levels of GRK2 (βARK1) Mirror Changes in the LVAD-Supported Failing Human Heart: Lower GRK2 Associated With Improved β-Adrenergic Signaling After Mechanical Unloading. J. Card. Fail..

[102] Agüero J., Almenar L., D’Ocon P., Oliver E., Monto F., Rueda J., Vicente D., Martínez-Dolz L., Salvador A. (2009). Myocardial and Peripheral Lymphocytic Transcriptomic Dissociation of β-adrenoceptors and G Protein–coupled Receptor Kinases in Heart Transplantation. J. Hear. Lung Transplant..

[103] Rengo G., Galasso G., Femminella G.D., Parisi V., Zincarelli C., Pagano G., De Lucia C., Cannavo A., Liccardo D., Marciano C. (2014). Reduction of lymphocyte G protein-coupled receptor kinase-2 (GRK2) after exercise training predicts survival in patients with heart failure. Eur. J. Prev. Cardiol..

[104] Bonita R.E., Raake P.W., Otis N.J., Chuprun J.K., Spivack T., Dasgupta A., Whellan D.J., Mather P.J., Koch W.J. (2010). Dynamic Changes in Lymphocyte GRK2 Levels in Cardiac Transplant Patients: A Biomarker for Left Ventricular Function. Clin. Transl. Sci..

[105] Rengo G., Pagano G., Filardi P.P., Femminella G.D., Parisi V., Cannavo A., Liccardo D., Komici K., Gambino G., D’Amico M.L. (2016). Prognostic Value of Lymphocyte G Protein-Coupled Receptor Kinase-2 Protein Levels in Patients With Heart Failure. Circ. Res..

[106] Santulli G., Campanile A., Spinelli L., Di Panzillo E.A., Ciccarelli M., Trimarco B., Iaccarino G. (2011). G Protein-Coupled Receptor Kinase 2 in Patients With Acute Myocardial Infarction. Am. J. Cardiol..

